# Diet of land birds along an elevational gradient in Papua New Guinea

**DOI:** 10.1038/srep44018

**Published:** 2017-03-09

**Authors:** Katerina Sam, Bonny Koane, Samuel Jeppy, Jana Sykorova, Vojtech Novotny

**Affiliations:** 1 Biology Centre CAS, Institute of Entomology, Branisovska 31, 370 05 Ceske Budejovice, Czech Republic; 2 University of South Bohemia, Faculty of Science, Branisovska 1760, 370 05 Ceske Budejovice, Czech Republic; 3 The New Guinea Binatang Research Center, PO Box 604 Madang, Papua New Guinea

## Abstract

Food preferences and exploitation are crucial to many aspects of avian ecology and are of increasing importance as we progress in our understanding of community ecology. We studied birds and their feeding specialization in the Central Range of Papua New Guinea, at eight study sites along a complete (200 to 3700 m a.s.l.) rainforest elevational gradient. The relative species richness and abundance increased with increasing elevation for insect and nectar eating birds, and decreased with elevation for fruit feeding birds. Using emetic tartar, we coerced 999 individuals from 99 bird species to regurgitate their stomach contents and studied these food samples. The proportion of arthropods in food samples increased with increasing elevation at the expense of plant material. Body size of arthropods eaten by birds decreased with increasing elevation. This reflected the parallel elevational trend in the body size of arthropods available in the forest understory. Body size of insectivorous birds was significantly positively correlated with the body size of arthropods they ate. Coleoptera were the most exploited arthropods, followed by Araneae, Hymenoptera, and Lepidoptera. Selectivity indexes showed that most of the arthropod taxa were taken opportunistically, reflecting the spatial patterns in arthropod abundances to which the birds were exposed.

Our knowledge of species richness patterns along elevational gradients has increased considerably over recent decades, but our understanding of the underlying mechanisms shaping these patterns has not^1^. The relative importance (in terms of species richness and/or abundance) of individual feeding guilds, and trends in feeding preferences in avian communities along elevational gradients have rarely been studied^2^. However, feeding preferences and trophic niche partitioning might be important mechanisms shaping patterns of species richness along environmental gradients^3^,^4^. Although some aspects of feeding ecology of birds along gradients have been studied, including diet breadth^5^,^6^,^7^, diet overlap^8^, and diet spatial fluctuation^9^,^10^, rigorous community-wide comparison of dietary specialization of birds along elevational gradient and its importance for niche theory have appeared only recently^3^,^4^.

In Peru, diverging patterns of species richness along environmental gradients were described for individual feeding guilds. Insectivores were dominant at low elevations, while frugivores at higher elevations in bird communities^11^. On the other hand, insectivores remained the dominant guild along entire elevational gradients in Costa Rica^12^, the Himalayas^3^ and Papua New Guinea^13^. The insectivores at the highest elevations represented 62–66% of their lowland species richness in Peru and Costa Rica, but only 35% in Papua New Guinea, and they were species richer at high than at low elevations in the Himalayas. It has been hypothesised that reduced syntopy among insectivores at high elevations is a response to simplified vegetation^11^,^14^, lower habitat complexity^13^ or an altered food resource base^3^,^13^.

The availability and exploitation of food resources are crucial to many aspects of avian ecology, and it is increasingly important that we understand their impact on bird community ecology and organization. Yet, quantitative studies of food availability and food exploitation by birds remain rare (e.g. refs 3 and 15). The diet of tropical bird species is poorly known^16^,^17^,^18^, and rarely confronted with available food resources (but see ref. 3).

Prey size is another important factor in prey choice^19^,^20^,^21^,^22^,^23^,^24^. Prey size is usually positively correlated with predator size in birds^20^,^25^,^26^,^27^, including insectivorous birds^20^,^28^,^29^. Thus the size distribution of prey may influence the distribution and survival of individual bird species and affect the structure of avian guilds^9^,^30^. Schoener^31^ found that large-billed insectivorous birds, which are predators of relatively large prey, were absent at sites where large-bodied prey was rare. Janes^20^ found that body size of foliage-gleaning birds was correlated more closely with arthropod size than the elevation. The size-related feeding preferences were never studied directly on large scales, and the predator-prey relationship between birds and insect have rarely been investigated along elevational gradients.

To redress these knowledge gaps, we quantified food availability and used emetic tartar to estimate the actual diet of birds along an elevational forest gradient in Papua New Guinea. We tested the following predictions: (1) species richness of insectivorous birds will decrease faster than species richness of other feeding guilds^11^ (2) relative importance of insectivorous birds within a community decreases with increasing elevation^11^, (3) specialization of insectivorous birds decreases with increasing elevation, (4) body size of insects and birds are correlated along the gradient, and decrease in parallel with increasing elevation^20^, and (5) diet of birds reflects trends in arthropod abundance along the elevational gradient.

## Results

We coerced 999 birds from 99 species mist-netted at eight sites along the altitudinal gradient to regurgitate their stomach contents. Eighty-five birds (8.5%) regurgitated only liquid, and 170 birds (17%) failed to regurgitate. We thus obtained 744 food samples from 99 bird species, and identified 3,480 food items of which 2,730 items were arthropods (complete or their body parts) and the rest plants (seeds or plant fragments; Table S1). Overall, 78 bird species were represented by >3 food samples (35 species were represented by 4–5 samples, and 43 bird species by >5 samples; Table S1) and used in the subsequent analyses.

**Figure 1:**
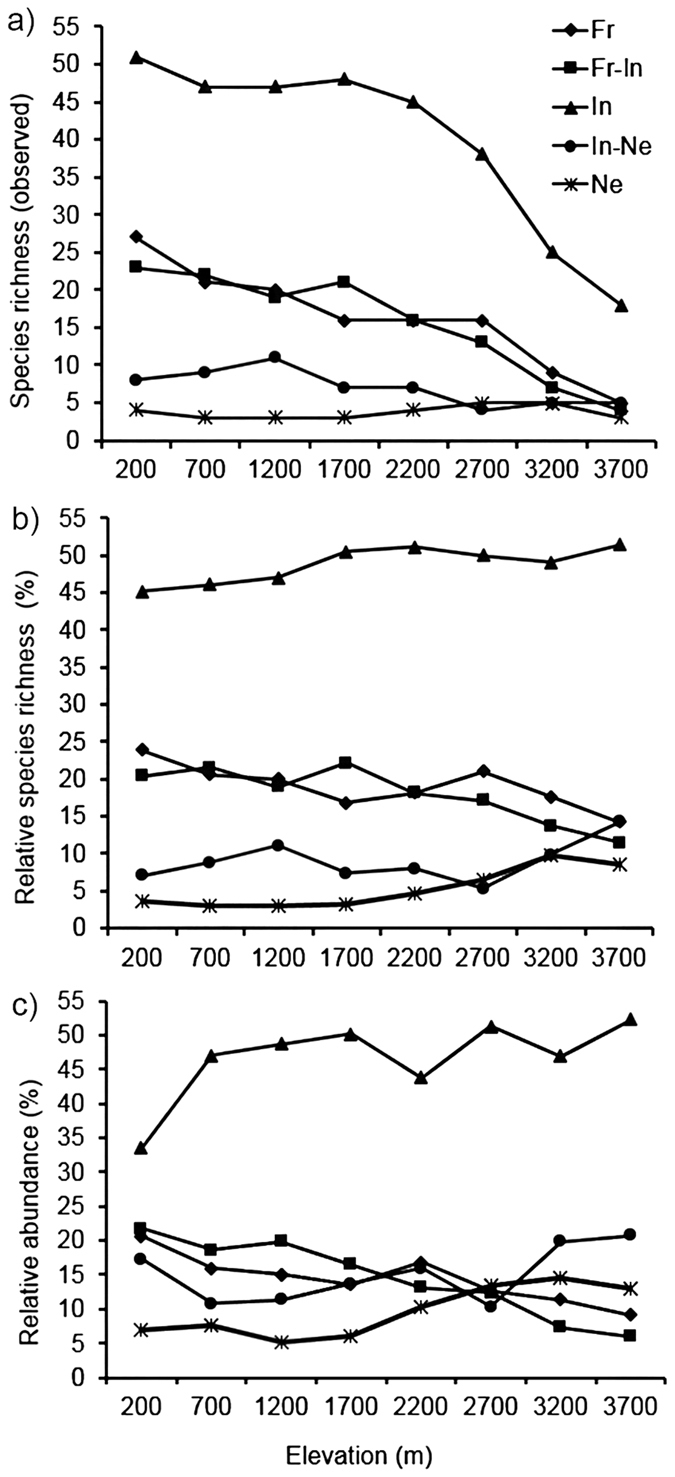
Species richness of birds along elevational gradient of Mt. Wilhelm, partitioned into feeding guilds based on literature (**a**). Relative species richness (**b**) and relative abundance of feeding guilds along the elevational gradient (**c**). Fr–frugivores, Fr-In–frugivore-nectarivores, In–insectivores, In-Ne–insectivore-nectarivores, Ne–nectarivores.

The whole avian community of Mt. Wilhelm included 236 bird species from which 49 were identified as frugivores (Fr), 46 as frugivore-insectivores (Fr-In), 112 as insectivores (In), 17 as insectivore-nectarivores (In-Ne), 11 as nectarivores (Ne) and one as granivore according to the literature (Table S3, Fig. 1a). We were able to further investigate and describe the intake of 12% of these frugivores, 22% of frugivore-insectivores, 47% of insectivores and 62.5% of insectivore-nectarivores. These numbers show that sampling was not balanced across the feeding guilds, and that we were more likely to mist-net insect-eating than fruit-eating birds. The proportions of individual guilds remained the same along the whole elevational gradient.

**Figure 2:**
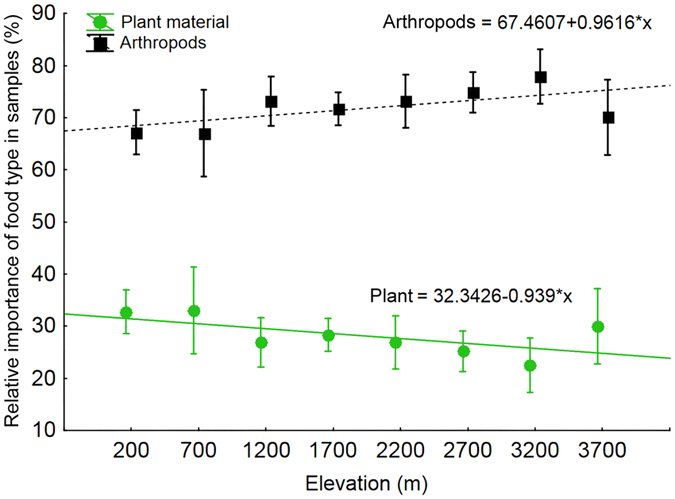
Relative importance of food types (plant material or arthropod’s body parts) in food samples of birds mist-netted in understory of Mt. Wilhelm elevational gradient.

Observational data showed that while the relative importance (i.e. the proportion of all bird species recorded per elevational study site) of insectivorous, insectivore-nectarivorous and nectarivorous guilds increased with increasing elevation, the relative importance of frugivorous and frugivore-insectivorous guilds decreased with increasing elevation (Fig. 1b,c). This result corresponded to the relative importance of plant and animal food items in combined samples, where we observed significantly decreasing (F_1,335_ = 4.63, p = 0.032, β = −0.012, Fig. 2) importance of plant material (fruits, seed and non-reproductive plant material) and significantly increasing (F_1,335_ = 4.634, p = 0.032, β = 0.017, Fig. 2) importance of animal material (mostly arthropods). However, somewhat different elevation trends in the importance of plant and animal material were observed within individual feeding guilds (Fig. S2).

Sampling of stomach contents was conducted during the wet and the dry season. This might have had an effect on the relative intake of arthropod versus plant matter, although the sites are tropical and there is very low seasonality breeding. We compared relative intake of food matter between the wet and dry seasons for bird species mist-netted in more than four individuals in each season at the same elevation (N = 21 species) to investigate potential confounding effects of seasonality. There was no effect of season on the proportion of plant and animal items in bird diet (Man-Whitney tests, Z = 0.31–1.77, P = 0.61–0.07).

**Figure 3:**
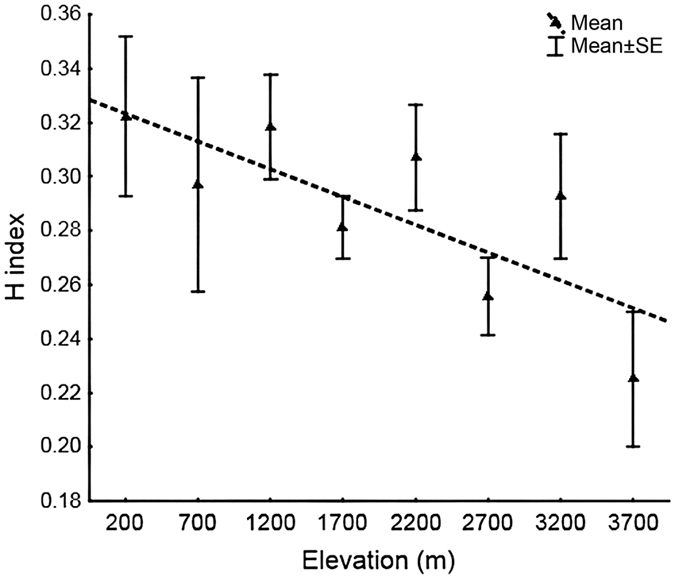
Brillouin diversity index (H) of invertebrate morphospecies in the diet of insectivorous birds along Mt. Wilhelm elevational gradient.

Diet varied considerably among conspecific individuals, and more samples would be necessary to describe food exploitation by individual species. About 10–30 samples would be sufficient to describe arthropod exploitation by some species, while we investigated an average of only 9 (range 4–35) samples. We based these estimates on visual investigation of observed and extrapolated food morphotype accumulation curves and corresponding Chao2 estimates for the more extensively sampled bird species (Fig. S3). The overall diversity of arthropod morphotypes in the food of well-sampled bird species (i.e. those with >10 samples including >6 food items) significantly decreased with increasing elevation (Mean H = 0.33 −0.02*Elevation, F_(1,228)_ = 5.424, P = 0.02, Fig. 3).

**Figure 4:**
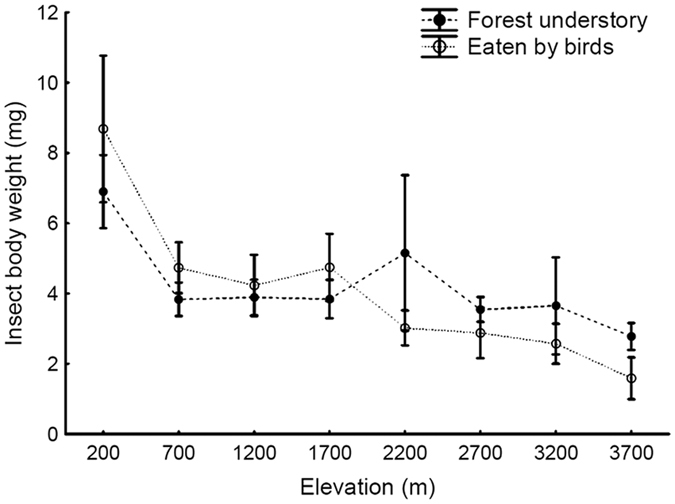
Arithmetic mean (±SE) of body weight (log grams) of insect collected in forest understory by insecticide spraying and found in food samples of mist-netted birds.

We obtained body length, extrapolated to body weight^32^, for 1,538 arthropods taken by 62 bird species from all sites (n = 185–123 arthropods/site). We also measured body size and calculated body weight of 8,204 arthropods occurring in forest understory of the study sites. The body size of arthropods present on foliage in the forest understory decreased with increasing elevation (Mean body weight (mg) = 5.7798–0.3509*Elevation (m), Fig. 4). The body size of arthropods present in the forest understory followed similar elevational trends to the body size of arthropods taken by birds (Mean body weight (mg) = 7.6047–0.7884*Elevation (m), Fig. 4). There was no significant difference in the body size of the arthropods that were eaten by birds and those present in forest understory (F_7, 8717_ = 0.12, P = 0.99; Fig. 4). The elevational trend was caused mainly by the body weight of Araneae, Coleoptera and Hymenoptera (without Formicidae) that decreased significantly with increasing elevation when we tested individual arthropod orders separately. Other orders (Neuroptera, Hemiptera and Diptera) did not show any trend or occurred only scarcely in the samples (n < 100 individuals, or n < 5 individuals/site). Ants (Formicidae) did not occur in samples at higher elevations.

**Figure 5:**
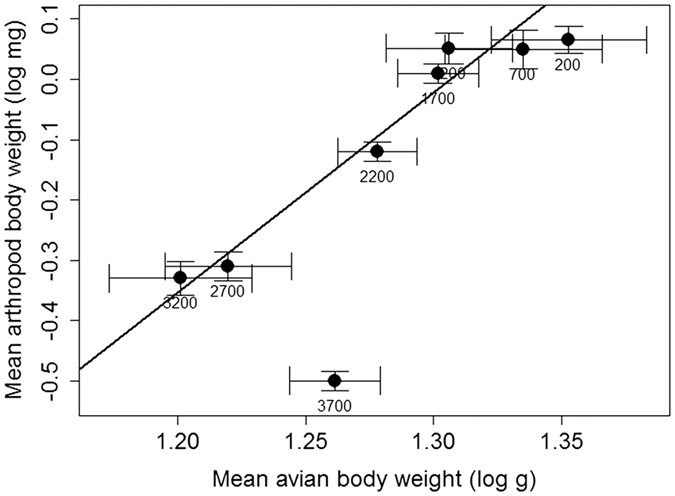
Relationship between mean log (body weight) of bird individuals and arthropod prey in their food samples. Each point represents one bird community. Mean arthropod weight = 3.3999*Mean avian weight −0.2407, R^2^ = 0.941, P > 0.001, F_1,7_ = 69.87. Note that data for 3700 m are not included in the regression.

The mean body weight of insectivorous bird species declined with increasing elevation from 21.03 g at 200 m a.s.l. to 15.88 g at 3200 m a.s.l., and 21.1 g at 3700 m a.s.l. (Mean bird weight (log g) = −0.0004*Elevation (m) + 1.342, R^2^ = 0.86, F_1,8_ = 43.21, p < 0.001). The mean weight of individual insectivorous birds in communities and their insect prey were correlated between elevations 200–3200 m asl (n = 9, R^2^ = 0.91, F_1,7_ = 67.83, p < 0.001, Fig. 5). Inclusion of an outlying data point from 3700 m made the correlation non-significant (n = 10, R^2^ = 0.18, F_1,7_ = 1.13, p = 0.328).

**Figure 6:**
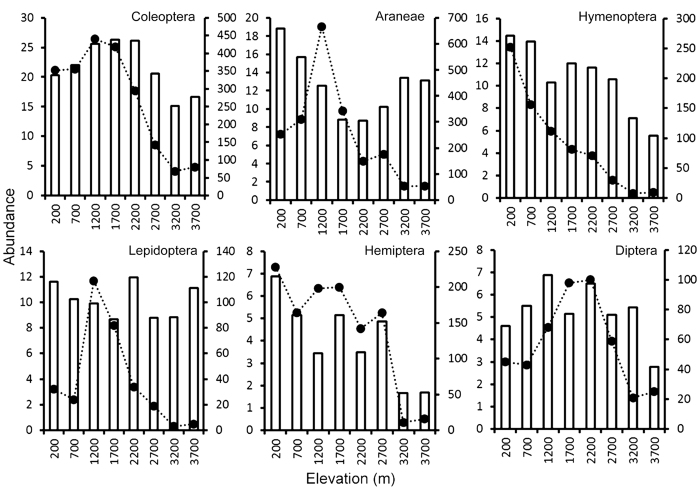
Abundance of arthropod taxa in samples collected by insecticide spray in forest understory (bars, right axis Y; standardized per 200 m^2^ leaf area per elevation) and in food samples (line, left axis Y; standardized per 100 food samples per elevation).

Coleoptera (713 individuals) was the best represented arthropod taxon (*sensu* Table S1) in bird diet, found in the diet of 90 bird species. It was followed by Araneae (399 individuals taken by 81 bird species), Hymenoptera (469 individuals, of which 201 were ants, taken by 80 bird species), and Lepidoptera (103 adults and 262 larvae taken by 64 bird species). These four invertebrate taxa accounted for 67% of all invertebrate items found in all food samples. Most sampled bird species fed on several taxa (mean = 6.3, Fig. 6) from the 21 broadly defined arthropod taxa (Table S1).

**Figure 7:**
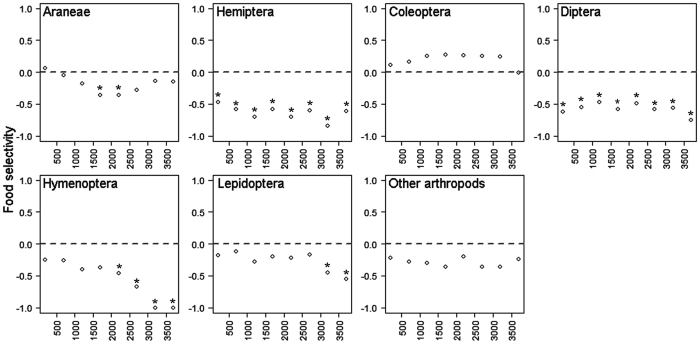
Prey selectivity (ϵ_*i*_) of forest understory birds. Positive values indicate preferred prey. Values significantly different from zero (P = 0.05, Holm’s correction of P-levels within each predator species was used) are marked by asterisk. ϵ_*i*_ index (sensu Chesson 1983^33^) can assume values between −1 and 1.

The abundance of arthropods in food samples reflected, more or less, their overall abundance in the forest understory, with Coleoptera (26% of all collected arthropods) being the most abundant, followed by Araneae (24%), Hemiptera (14%), Hymenoptera (9%), Diptera (5%) and Lepidoptera (4%). Hymenoptera and Hemiptera were more abundant in lowlands than at high elevations both in the forest understory and in food samples (Fig. 6), and preference (calculated against the background data from forest understory; see Methods and ref. 33) for them also decreased with increasing elevation (Fig. 7). Coleoptera and Diptera showed a hump-shaped pattern in abundance along the elevational gradient both in the forest understory and food samples. While Coleoptera was the most preferred food item overall, its preference peaked between elevations 1,200 and 3,200 m (Fig. 7). Diptera were not preferred prey at any elevation (Fig. 7). Araneae and Lepidoptera showed no clear pattern in abundance in food samples, and were the most abundant at lower-mid elevations in the forest understory. While birds showed clear preference for Lepidoptera at low elevations, Areneae were preferred both at the lowest and highest elevations (Fig. 7).

## Discussion

Our study brings the first report on food preferences of many bird species from Papua New Guinea. Our data are consistent with the hypothesis that species richness of insectivorous birds decreases faster (ca. 10 species per 1000 m) than species richness of other feeding guilds (ca. 6-1 species per 1000 m) with increasing elevation (Fig. 1a). This decrease was also faster when comparing Mt. Wilhelm to the Andean^11^ and Costa Rican^12^ elevational gradients (Fig. S4). However, the insectivores represent higher proportion of avian community at high elevations than at low elevations (Fig. 1b). We show that the diversity of food items taken by birds decreases towards higher elevations, and that the disappearance of some insect taxa from avian diet corresponds with their disappearance from the pool of available food resources. Our findings support the notion that the body-sizes of insectivores are to some extent determined by the insect size composition. To our knowledge, this is the first study bringing detailed description of the food preferences of variety of bird species along a complete elevational gradient.

The insectivorous birds along Mt. Wilhelm represented a higher proportion of the avian community at higher elevations (52% at 3700 m asl) than in lowlands (45% at 200 m asl). Terborgh^11^ found opposite pattern, where insectivores represented 64% of avian community in lowlands (ca. 500 m) and only 40% at 3600 m asl. (Fig. S4). Blake and Loiselle^12^ observed a similar decrease in insectivores with elevation along a shorter gradient (Fig. S4). These differences may be explained by the environmental conditions along the gradients or the composition of avian communities (e.g., there are about three times more nectarivorous birds along the Andean than along Mt. Wilhelm gradients). The total species richness also decreases two times faster along South American gradients than along Mt. Wilhelm.

The relative species richness of feeding guilds corresponded to overall trends in the importance of food types in food samples. In food samples, we observed plant material to be relatively more important at low elevations than at high elevations for all birds together and most of the feeding guilds. We did not detect any difference between sampling seasons. We did not observe any significant change in preferences of food types by insectivorous birds along the gradient or between seasons. This implies that the overall pattern is driven by fruit-eating birds that are taking many more insects at higher elevations than in the lowlands (Fig. S2). Such patterns have not been described and discussed before. However, Craig *et al*.^34^ reported that honeyeaters occurring in lowlands (0–300 m) eat much more nectar and fruits than arthropods (10:1 ratio), while similar sized honeyeater with much wider elevational range (0–1220 m) feed more on arthropods (1:1.8 Nectar + Fruits: Arthropods ratio).

Diversity of arthropod morphospecies in food samples decreased with increasing elevation in the food samples of insectivorous bird species. This result might reflect a general decrease in the species richness of most arthropods with increasing elevation^35^,^36^,^37^, or disappearance of some arthropod taxa at high elevations^11^. Simplification of the habitat might be another explanation. Robinson and Holmes^10^ suggested that certain habitat characteristics, particularly the physical structure of the habitat, have been important selective forces in determining the foraging behaviour and resource exploitation. As the complexity of habitat decreases with increasing elevation along the Mt. Wilhelm gradient, the birds might be forced to employ a less diverse range of foraging tactics and catch less diverse groups of arthropods in higher elevations as a result. Incompleteness of our sampling, and potentially missing information on food item variability has to be taken into account when we try to interpret these results.

The body size of arthropods present in forest understory correlated with the body size of arthropods taken by birds, and body sizes of all arthropods decreased with increasing elevation. The mean body size of arthropods is known to decrease from leaf-litter to the canopy^38^, and from lower to higher elevations^36^,^39^,^40^,^41^. As we sampled mostly birds feeding in the forest understory, together with understory arthropods, it is not surprising that we found close correlation between the body sizes of arthropods present in the forest and in the food samples. If large-bodied prey were more profitable than smaller prey, it would pay off to focus on prey with a large body size whenever possible^42^. Our unpublished results from a different experiment conducted at the same study sites showed that the removal of insectivorous birds from the forest system led to an increase of the mean body size in arthropod community. Specifically, mean body size of arthropods in communities where birds were excluded for 6 months increased from 4.1 mm to 5.6 mm at 200 m asl, and a similar increase in body size was evident at all elevations up to 2700 m asl (Sam *et al*., unpubl. data). This suggests that even though the body sizes may be sampled proportionately by birds, the impact of predation on the populations of large-bodied arthropod species is particularly significant.

The body size of individual birds is correlated with the size of the insects taken. Our results mirror findings of Price^43^ who documented strong correlation between body size of warblers and head size of beetles found in their faeces. This was suggested as a general pattern^20^ for avian guilds dependent directly or indirectly upon arthropods, but not for other guilds. This might explain why the 3700 m study site was an outlier in the general bird–insect prey correlation of body size. Birds present there were usually large bodied species taking small insects. None of those species were specialized insectivores. Most of them feed on nectar and fruits, and foraged mainly in the forest canopy. They seemed to take frugivorous insects together with fruits, or accidentally when reaching for nectar.

We did not observe any strong preferences by birds for the studied arthropod taxa, and most of the preference indexes indicated that birds were taking food items rather randomly. Coleoptera were overall the most abundant food item followed by Araneae, Hymenoptera and Lepidoptera. This reflected observed abundances of these groups at our study sites, as well as in other tropical forests^44^,^45^,^46^. Diptera were clearly the least favoured food (i.e. abundant in samples from the forest understory but not in food samples). It might be a product of the foraging methods of the surveyed birds, as most of them were gleaners and Diptera are usually caught in flight.

Various techniques are usually needed to survey food resources available to birds, and good ecological knowledge on avian feeding techniques is needed to discuss the availability and exploitation of food in detail. In the case of our study, we sampled only arthropods from the foliage of the forest understory, and we assumed that most of the mist-netted birds also forage there. We might miss some actively flying arthropods which are usually hunted by flycatchers; these would be more appropriately monitored by Malaise and flight intercept traps. In the view of these approximations, our data on food offer and food exploitation correspond surprisingly closely with each other for most taxa and elevational study sites.

The presence of ants in food samples, for example, reflects nearly perfectly their distribution along our elevational gradients, and their steep decrease in abundance with elevation. Based on pitfall trapping (Fayle and Moses, unpubl. data) and tuna baits (Sam, unpubl.data), ants were highly abundant at low elevations but became scarce at 2200 m and not occurring at 2700 m and higher. Birds having ants in their food samples were observed searching mainly in the understory (95% of eaten ants were workers). This observation corresponds with findings of Sherry^47^, who used frequencies of aerial vs. non-aerial foraging tactics to determine whether flying (reproductive) or non-flying (primarily worker) ants were eaten by Neotropical Flycatchers.

An exact assessment of bird diet would be a difficult task, and we have to keep in mind that we present only data on food taken by nearly 1000 individuals of 100 bird species foraging mostly in the understory of tropical forest. More extensive surveys using multiple techniques (including for instance canopy insecticide fogging, pitfall, Malaise and flight intercept traps) would be needed to estimate available food resources more precisely, across all forest strata. However, such data is rare and difficult to obtain.

## Methods

The study was carried out at eight study sites in Papua New Guinea. Sites were regularly spaced, from 200 to 3700 m a.s.l. with 500 m elevational increments along an elevational gradient of Mt. Wilhelm (−5.44, 145.20; −5. 47, 145.03). Average annual precipitation is 3288 mm (local meteorological station) in the lowlands, rising to 4400 mm at 3700 m a.s.l., with a distinct condensation zone around 2500–2700 m a.s.l.. Mean annual temperature decreases from 27.4 °C at the lowland site to 8.37 °C at the tree line at a constant rate of 0.54 °C per 100 elevational metres. The habitats within the surveyed transect could be described as lowland alluvial forest (200 m a.s.l.), foothill forest (700 and 1200 m a.s.l.), lower montane forest (1700–2700 m a.s.l.), and upper montane forest [3200 and 3700 m a.s.l.; according to ref. 48]. The typical species composition of forest^48^ and general climatic conditions are described elsewhere^13^,^49^. We confirm that all experiments on vertebrates mentioned here were performed in accordance with relevant guidelines. We mist-netted the birds according to Czech and Australian guidelines (licenses CZ1062 and ABBBS No. 3173). The experiments were approved and performed under guidelines of University of South Bohemia in the Czech Republic and its experimental protocol 1515-20424/2010-16 with extension 1515-20424/2012-30.

### Bird community survey

Bird communities were surveyed using point counts and mist-netting. The sampling effort is described in detail by refs 13 and 50. Briefly, point counts were carried out at 16 points regularly spaced along a 2400-m transect (successive points were 150 ± 5 m apart to avoid overlap). All birds seen or heard within a fixed radial distance of 0–50 m were recorded. We started censuses 15 min before sunrise and finished before 11:00. We replicated all 16 point-counts 14 times (during 14 days) at each study site.

We mist-netted birds using 200 meters (2.5 × 18 or 12 m, 16 mm mesh) of nets per site. At each study site, we opened mist-nets for three days (from 05:30 to 17:30 Standard time, mist-nets visited in 20-min intervals) during wet season and five days during dry season. All birds were captured, weighed, measured, identified to species and sex (where possible), banded with a colour ring and forced to regurgitate. Three people performed these procedures (KT, BK, SJ). All birds were released within 10 minutes. Bird surveys along the elevational gradient were conducted between 9^th^ April and 31^th^ May 2010, 26^th^ July and 15^th^ November 2010, and between 15^th^ May and 15^th^ October 2012, visiting thus each study site both during wet season (Apr–May and Oct - Nov) and dry season (Jun - Sep).

### Food intake sampling

Food samples were obtained by administering tartar emetic following Poulin *et al*.^51^,^52^,^53^. Immediately after capture, birds were given 0.8 cm^3^ of 1.5% antimony potassium tartar per 100 g of body mass. Concentration 1.0% was used for birds smaller than 10 g^52^. The solution was given orally through a flexible plastic tube attached to a 1-cc syringe. After administration, the birds were placed in a special “regurgit-bowl” (Fig. S1). The bottom of regurgit-bowl was cleaned with water, detergent, and toilet tissue after each bird. Regurgitated food items were preserved in absolute ethanol (99%). Weak individuals found in the net and breeding females were not subjected to regurgitation. All individuals were sampled only once, and were released all recaptured individuals immediately. Bird mortality was only 0.2% (i.e., two individual deaths during handling). We did not observe any significant differences in mark-recapture rates between sites when emetics were used and those at which they were not.

We (KT, JS) examined each food sample (defined as regurgitated food of a single bird individual) under a dissecting scope. The minimum number of arthropod individuals per morphospecies was determined from body part counts in the sample. Most of the arthropods were fragmented, and their identification was thus based on the least digestible and most characteristic parts (guide available online http://tvardikova.weebly.com/downloads.html). Individual arthropods were identified to morphospecies (i.e., morphologically identical prey categories assumed to represent one species), and classified to order or family where possible. Further analyses were also based on the classification of arthropods into higher taxa (as listed in Table S1). We used published information^54^,^55^ and the insect collections of our team as reference. We measured the length of each arthropod individual or body part to the nearest 0.1 mm. We estimated the body length according to the published order-specific equations using the lengths of different body parts^56^,^57^,^58^. We used body length to estimate body weight for arthropods according to Ganihar^32^. Presence or absence of nectar, and its relative volume in sample, was evaluated through the presence of pollen grains using microscope^59^. Similarly, percentage of plant material was estimated from the volume of plant parts. Minimal number of fruit was estimated (in cases when a sample contained multiple small seeds that could all have come from a single fruit) or counted.

Each food sample contained the remains of food eaten over an unspecified but limited time, and as such does not represent the complete diversity of food items taken. We calculated food specialization using the Brillouin diversity index (H) according to Sherry^47^, and examined the cumulative curves for all food species to identify whether our sampling effort was sufficient. We calculated selectivity index for insect taxa according to Chesson^33^. We used index ᾳ (equation 2, Chesson^33^), which predicts no deletion of food resources and takes into account available and consumed abundances of food items of a given type. For display purposes only, we calculated index ϵ (equation 15, Chesson^33^) which ranges between −1 and 1 irrespective to food densities. For statistical analyses, ᾳ has to be used as ϵ has no statistical properties given.

### Arthropod sampling

To sample arthropods at each elevational study site, we carefully lowered crowns of 20 saplings (3–5 m high) of several tree species (listed in Table S2). We wrapped the whole sapling crowns into a mosquito net and fumigated them with fast knock-down insecticide (Mortein^®^). Individual saplings were about 50 m apart, and their crowns had 12.2 ± 2.3 m^2^ of foliage. After fumigating, we shook the foliage thoroughly in the mosquito net, collected all dislodged arthropods into ethanol filled vials, and visually inspected the foliage for any remaining individuals. Individual arthropods were measured and identified to orders or other groups (as listed in Table S1). The arthropods of forest understory were surveyed between 9^th^ April and 15^th^ June 2014, and between 26^th^ July and 15^th^ November 2014. Logistics of the project did not allow sampling of the insects in same years as sampling of food intake. However, to confirm that the food offer is stable (on the taxonomic level we used in this work) over the years, we sampled one elevational study site (200 m a.s.l.) both in wet and dry season 2010, 2012 and 2014.

## Additional Information

**How to cite this article:** Sam, K. *et al*. Diet of land birds along an elevational gradient in Papua New Guinea. *Sci. Rep.* **7**, 44018; doi: 10.1038/srep44018 (2017).

**Publisher's note:** Springer Nature remains neutral with regard to jurisdictional claims in published maps and institutional affiliations.

## Supplementary Material

Supplementary Materials
