# Supplementary material for: Diet of land birds along an elevational gradient in Papua New Guinea
Source: Sci Rep. 2017 Mar 9;7:44018. doi: 10.1038/srep44018 (PMC5343654; doi:10.1038/srep44018)

SUPPORTING INFORMATION

Diet of land birds along an elevational gradient in Papua New Guinea

Katerina Sam, Bonny Koane, Samuel Jeppy, Jana Sykorova, and Vojtech Novotny

Figure S1. Regurgit bowl – picture of the inner side with a food sample in it (left), hanging with birds inside (right)

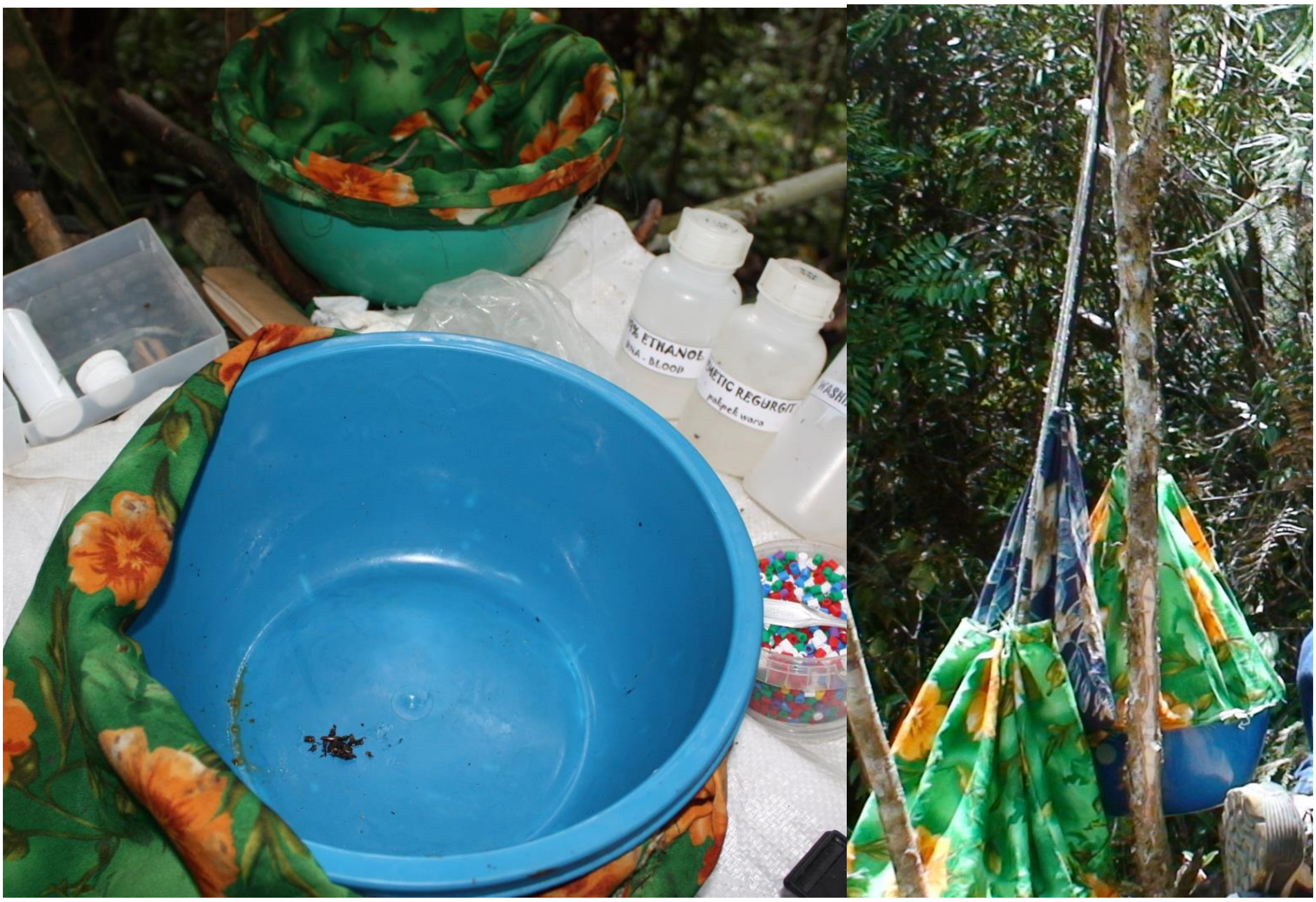

Table S1. Abundance of different food types in the diet of bird species. All 100 bird species represented, and species represented by ≥4 food samples are marked with asterisk and indented. Total number 774 successfully obtained samples are summarized here. The following miscellaneous items are not included in the table: fish and small rodent fragments in the samples *of Ceyx lepidus* (n=3), and bones of lizards in the samples of *Colluricincla megarhyncha* (n = 3), *Peneothello cyanus* (n = 2), *Grallina bruijni* (n = 2), *Pitohui dichrous* (n = 1), *Pachycephala hyperythra* (n = 1), and *Tregellasia leucops* (n = 1). One sample of *Alcedo azurea* included a nearly complete crab specimen (*Brachyura*). Small stones were found in samples of many species taking larger insects and/or seeds (e.g. *Colluricincla megarhyncha*, *Ifrita kowaldi*, *Grallina bruijni*), and in samples from all surveyed kingfishers.

Bird Species	Number of samples	Number of invertebrate taxa	Areneae	Chilopoda	Coleoptera	Dermaptera	Diplopoda	Diptera	Gastropoda	Neuroptera	Odonata	Orthoptera	Ricinulei	Hemiptera	Lepidoptera adult	Lepidoptera larvae	Hymenoptera: ants	Hymenoptera: others	Hymenoptera: bees	Hymenoptera: wasps	Insect:egg	Insect: larvae	Insect: pupae	Nectar	Fruit (+Seeds)	No. of invertebrates or fruits obtained per species	Mean number of food items in sample
Acanthiza murina	4	5	2		<u>4</u>				1						1	1	3	2			1			Yes	2	17	4.3
Alcedo azurea	10	3	5	1		2	2		5	4	8	5		<u>10</u>		2										44	4.4
Aleadryas rufinucha	4	5	2		3					1			1		2	3		1			4				4	21	5.3
Amalocichla incerta	7	6	2		<u>8</u>			2	1					<u>6</u>		<u>6</u>		4		2				Yes	2	33	4.7
Arses insularis	5	4	4		2									1	3			<u>5</u>			4				4	23	4.6
*Cacomantis castaneiventris**	2		2		2											3	1					1				9	4.5
Ceyx lepidus	4	10	7		<u>8</u>			4			3	1		3		2	2	1	1		3				4	39	9.8
*Chaetorhynchus papuensis**	2				1			1						<u>4</u>	1	1	1				2					11	5.5
Chalcophaps stephani	5	3			2			1									1								26	30	6
Clytomyias insignis	4	5			2			1							1	3		2							1	10	2.5
Cnemophilus macgregorii	1	1															1							Yes	7	8	8
Colluricincla megarhyncha	17	8	10		<u>11</u>			1	2		1	1		3		<u>15</u>	4				6		6	Yes	9	69	4.1
Coracina melas	4	5	1		2			1					1			1	1								1	8	2
Coracina montana	10	2	<u>8</u>		2					5	1	<u>8</u>					1				3					28	2.8
Crateroscelis murina	11	12	10	2	<u>19</u>		1	2				1		4		9	8	5			<u>11</u>				6	78	7.1
*Crateroscelis nigrorufa**	1		1					1						1	1	2		1			1			Yes		8	8
Crateroscelis robusta	22	11	15	2	<u>37</u>			5					1	6		16	<u>17</u>	13	1	1	3	6	9	Yes	15	147	6.7
Dacelo gaudichaud	4	1		5	3		2																			10	2.5
*Dicrurus bracteatus**	1		1		2										1	1					1					6	6
*Euaegotheles insignis**	1		1												1	1					1					4	4
*Eugerygone rubra**	1		1		1										1	1										4	4
*Eulacestoma nigropectus**	1				2									1												3	3
Gallicolumba beccarii	4	2	1		1			1																	24	27	6.8
*Gallicolumba rufigula**	1																								5	5	5
Garritornis isidorei	10	3	<u>8</u>		6							5				1		5							15	40	4
Gerygone chrysogaster	4	9	3	1	4			1				1		1		<u>4</u>	1	1							1	18	4.5
*Gerygone cinerea**	1		1		2									2				1								6	6
*Grallina bruijni**	1				1																				8	9	9
Ifrita kowaldi	4	10	3	1	<u>10</u>		2	3								2	1	2		1					4	29	7.3
*Lichenostomus obscurus**	2		1		2				1								1									5	2.5
Lonchura tristissima	4	1															1								17	18	4.5
*Melampitta lugubris**	1				4											3							2			9	9
Melanocharis nigra	20	6			<u>25</u>					1		2						2					2	Yes	58	90	4.5
*Melanocharis nigra**	1		5		6									1	3	3	3	2			6			Yes	26	55	55
Melanocharis striativentris	9	3			2											1		1			4				28	36	4
Melanocharis versteri	35	8	<u>14</u>		<u>10</u>			4						2	3	3	4	4			6		6	Yes	56	112	3.2
Melidectes belfordi	4	6	1		2			1		1						2		2			3				2	14	3.5
Melidectes fuscus	14	6	<u>10</u>		7			1						2		5		3						Yes	10	38	2.7
Melidectes princeps	13	2	5					3								1								Yes		29	2.2
Melilestes megarhynchus	5	7	<u>17</u>		4	1		1						2		6	6							Yes	5	42	8.4
Meliphaga analoga	18	5	<u>14</u>		5				1				1			4	2	1			5			Yes	40	73	4.1
*Meliphaga aruensis**	1													1								1		Yes		2	2
*Meliphaga montana**	1		1		<u>7</u>			4				1		1		4	1				4				3	6	6
Melipotes fumigatus	8	5	3		2			1									1		1						14	22	2.8
Microeca papuana	11	10	4		<u>21</u>	3	1				1	1		9		6	9	9		1	8				6	79	7.2

Micropsitta pusio	10																							Yes	24	24	2.4
Monarcha axillaris	6	6	3		7			2	1			1		3		2		4			6				3	32	5.3
*Monarcha frater**	1		1		1																1	1				4	4
Monarcha guttula	14	8	16		20			1			1			8	1	9	4	4			5		5		10	84	6
Monarcha manadensis	4	7	2		2			1						1		2	2	2			3				1	16	4
Myiagra alecto	10	7	1	1	5	2		1									2	1		1	3				3	20	2
*Myiagra cyanoleuca**	1		2		2									1			2	1			1					9	9
Myzomela rosenbergii	4	7	3		4			1					1	1			4	1			2			Yes		17	4.3
Oedistoma iliolophus	4	5	3		3											1	1	2			5			Yes	2	17	4.3
Pachycephala hyperythra	8	6	4		11			1								2	2	2			5				7	34	4.3
Pachycephala modesta	4	10	1		4			1		1				1		3	1	1		1	2	1			2	19	4.8
Pachycephala rufiventris	4	10	5		13			2					2	4	2	2	1		1				9		3	44	11
Pachycephala schlegelii	16	12	10		23	1		3		1		1	1	4	7	10	5	6					16		17	105	6.6
Pachycephala simplex	4	4	1		2												1	1							1	6	1.5
Pachycephala soror	4	4	1		5											4		1			4				3	18	4.5
Pachycephalopsis poliosoma	10	0			1			1													3				22	27	2.7
Paramythia montium	13	4			1									2		1		1			2				19	26	2
Peneothello bimaculata	10	11	4	1	19	1		6				1			4	6	7	1		1			6		6	63	6.3
Peneothello cyanus	22	8	6		31	1		2	1				1	2		5	8	10				4			18	89	4
Peneothello sigillata	20	11	17	1	35	1		6		1			1	2	1	24	1	3				1			13	107	5.4
*Philemon meyeri**	1		2		1																	4	1			8	8
Pitohui dichrous	4	5	4		6	1		1								1	3					1	2		6	25	6.3
Pitohui kirhocephalus	10	7	2		3			1				1		1	2	2	1						3		8	24	2.4
Pitohui nigrescens	10	1	2		5					2		2	1	8			9								9	38	3.8
Poecilodryas albispecularis	6	6	3		8									3		3	3	2			5				6	33	5.5
Poecilodryas hypoleuca	4	7	7		6					1				2	2	2	3	4			7				2	36	9
*Ptilinopus pulchellus**	1		1		1									1	1			1								5	5
Ptiloprora guisei	4	5	2		4											3		2			8	1			8	28	7
Ptiloprora perstriata	25	11	17		16	1		5				1	1	2	3	8	3	8	2	2	11			Yes	17	97	3.9
Ptiloris magnificus	6	8	3	1	5			2						1		4	1	4			8				10	39	6.5
Ptilorrhoa caerulescens	10	8	2		1	1		1						1		1	2		1						8	18	1.8
Rhagologus leucostigma	9	7	1		9	1								1	3	3	2	4			3		2		18	47	5.2
*Rhamphocharis crassirostris**	1				2			1																		3	3
Rhipidura albolimbata	16	8	5		24			4						5	2	2	6	24		1	3		11		6	93	5.8
Rhipidura atra	34	13	17	1	54	1		22				1		11	2	12	12	39		2			10		19	203	6
Rhipidura brachyrhyncha	6	7	3		3	1		3		1				1	7		3	4			3		6		3	38	6.3
*Rhipidura maculipectus**	1		1		3									1	1	1	4	1								12	12
Rhipidura rufidorsa	10	4	8		10							2			8		5	8								41	4.1
Rhipidura rufiventris	4	6	2		6			1							1		3	2			2				1	18	4.5
Rhipidura threnothorax	4	10	6		13	1		10		2		1		1	2	3	9	1			5		5		6	65	16
Sericornis arfakianus	4	4	1		2										2	3		1							3	12	3
Sericornis nouhuysi	18	9	10		22			1	1	1		1		2	2	5	2	7					19		12	85	4.7
Sericornis papuensis	8	8	6		7			1		1					7	5	1	1			4				6	39	4.9
Sericornis perspicillatus	22	11	20		36		1	7				1		5	2	7	4	18					2		19	122	5.5
Sericornis spilodera	4	7	2		11	1		1						3	4	6	1						5		3	37	9.3
Sericornis virgatus	4	8	3		3			3		1					1		2	1					8		1	23	5.8
Syma torotoro	10	5	1		3			1						2				1					3			11	1.1
Tanysiptera galatea	8	5	5		9			1	1		1			1	4		1	1			3		3		4	34	4.3
Toxorhamphus novaeguineae	11	7	14		10	1		4						2	5	5	5	1			4			Yes	9	60	5.5
Toxorhamphus poliopterus	5	8	4		8			1				1		1	4		1	2			2		5	Yes	5	34	6.8
Tregellasia leucops	4	6	3		6									1	3	2	3	2			3		1		3	27	6.8
Turdus poliocephalus	5				3																				12	15	3
Xanthotis polygrammus	5																							Yes	13	13	2.6
Zoothera heinei	4	3	1												1		1					1			1	5	1.3
Zosterops novaeguineae	4	5	1		3			1							1	2	1	1				1			1	12	3
Number of arthropods, fruits			397	17	718	21	9	139	15	24	16	40	12	144	100	260	199	246	7	13	183	23	147		750		
No. of bird species taking food			80	11	88	17	6	54	10	15	7	22	11	51	39	63	62	61	6	10	46	12	25		75		

Table S2. Tree species surveyed for arthropod communities at elevational study sites

	200	700	1200	1700	2200	2700	3200	3700
Ficus arfakensis	X	X	X	X				
Ficus badiopurpurea		X						
Ficus congesta	X							
Ficus conocephalifolia	X	X						
Ficus endochaete			X		X	X		
*Ficus hahliana**	X	X	X	X	X	X		
Ficus hispidioides	X							
*Ficus hombroniana*ǂ			X	X	X			
Ficus iodotricha				X	X	X		
Ficus mollior				X				
Ficus saccata					X	X		
Ficus subcuneata		X						
Ficus trichocerasa•	X	X	X	X				
Ficus wassa#					X			
Macaranga melanosticta							X	X
Myrsine papuana							X	X
Myrsine womersleyi							X	X
Pittosporum berberidoides							X	X
Number of species	6	6	4	6	6	4	4	4

**F. hahliana* is confirmed as a good species from 200-1,200 m of our elevational gradient. After this (1,700m-2,700m) a close relative/sister species occurs. However, this potential split was discussed only recently based on molecular differences. We were not able to distinguish the two species in the time of our experiment.

ǂ *F. hombroniana* is found between 200-1,200m. There are a few individuals at 1,200 m but most individuals from 1,700 m classified as *F. hombroniana* are probably *F. ihuensis*.

• *F. trichocerasa* has two sub-species along the elevational gradient. *F. trichocerasa* subsp. trichocerasa occurs between 200-1,700 m and *F. trichocerasa* subsp. pleioclada occurs between 1,700 m and 2,200 m. They co-occur at 1,700 m, and both subspecies were included in our study as they are difficult to distinguish in the field at 1,700m.

F. wassa comprises many varieties along the elevational gradient of Mt. Wilhelm. The variety included in our study was *F. wassa* var. nubigena which occurs along the gradient from 1,300 to 3,000 m.

Table S3. Literature based feeding specialization of bird species occurring along Mt. Wilhelm elevational gradient. References mentioned in comments: (1) = Hoyo d. J., Elliott J., Sargatal J. & Christie D. A. (1992-2011) *Handbook of the Birds of the World (vol. 1-16)*. Lynx Editions, Barcelona, Spain. (2) Beehler, B. M., Pratt, T. K. & Zimmerman, D. A. 1986. Birds of New Guinea. Princeton Univ. Press. (3) Freeman B. G. & Freeman A. M. C. (2014) Rapid upslope shifts in New Guinean birds illustrate strong distributional responses of tropical montane species to global warming. *Proceedings of the National Academy of Sciences* **111**, 4490-4. (4) Personal communication Andrew Mack. Feeding guilds: Fr – frugivores, Fr-In – frugivore-nectarivores, In – insectivores, In-Ne – insectivore-nectarivores, Ne – nectarivores, Gr – granivores.

Species (Scientific)	Species (English)	Comments	Guild Lit.	Food survey	Number of samples including:				Total	Guild Observed	Guild confirmed
					Fr	In	Fr-In	Ne-In			
Acanthiza cinerea	Grey Thornbill	Insectivorous, but no details of prey (1, 2).	In								
Acanthiza murina	New Guinea Thornbill	Primarily insectivorous. Seeds, fruit and flowers found in gizzard (1). Insectivorous (2).	In	Yes		1	2	1	4	Fr-In	No
Aegotheles albertisi	Mountain Owlet-nightjar	Diet consist of insect (1).	In								
Aegotheles insignis	Feline Owlet-nightjar	Insectivore (1).	In								
Aepypodius arfakianus	Wattled Brushturkey	Fallen fruit and seeds, probably also insect (1).	Fr								
Ailuroedus buccoides	White-eared Catbird	Fruits and insect (1). Frugivore (2)	Fr-In								
Ailuroedus melanotis	Spotted Catbird	Omnivorous, but predominantly eating a variety of fruits (1). Frugivore (2).	Fr-In								
Aleadryas rufinucha	Rufous-naped Whistler	Insectivore (1, 2)	In	Yes		4			4	In	Yes
Alisterus chloropterus	Papuan King Parrot	Takes fruits, seeds, berries and nuts (1).	Fr								
Alopecoenas beccarii	Bronze Ground Dove	Frugivore (1,2)	Fr	Yes			4		4	Fr-In	No
Alopecoenas jobiensis	White-breasted Ground Dove	Fruits and seeds, with some insects (1).	Fr								
Amalocichla incerta	Lesser Ground Robin	Insectivore (1).	In	Yes		4	2	1	7	Fr-In	No
Amblyornis macgregoriae	MacGregor's Bowerbird	Frugivore (1, 2).	Fr								
Anthus gutturalis	Alpine Pipit	Mainly small insects and their larvae, and other arthropods; grass seeds, berries and green herbaceous matter also recorded (1).	In								
Aplonis cantoroides	Singing Starling	Fruit, especially figs; also insects (1). Omnivore (2).	Fr-In								
Aplonis metallica	Metallic Starling	Omnivore (1, 2).	Fr-In								
Arses insularis	Ochre-collared Monarch	Insectivore (1, 2)	In	Yes		1	4		5	Fr-In	No
Artamus maximus	Great Woodswallow	Insectivore (1).	In								
Astrapia stephaniae	Princess Stephanie's Astrapia	Frugivore (1,2)	Fr								
Cacatua galerita	Sulphur-crested Cockatoo	Frugivore (1,2)	Fr								
Cacomantis castaneiventris	Chestnut-breasted Cuckoo	Insectivore (1, 2)	In								
Cacomantis flabelliformis	Fan-tailed Cuckoo	Caterpillars and other insect (1).	In								

Cacomantis leucolophus	White-crowned Cuckoo	Mainly insect, also fruit (1).	In									
Cacomantis variolosus	Brush Cuckoo	Insectivore (1, 2)	In									
Caligavis obscura	Obscure Honeyeater	Omnivore (1, 2).	Fr-In									
Caligavis subfrenata	Black-throated Honeyeater	Diet includes nectar, small arthropds and fruit (1). Nectarivore (2)	In-Ne									
Campochaera sloetii	Golden Cuckooshrike	Eats mostly fruit; insects also recorded (1). Frugivore (2)	Fr-In									
Caprimulgus macrurus	Large-tailed Nightjar	Insectivore (1).	In									
Carterornis chrysomela	Golden Monarch	Largely small invertebrates (1).	In									
Casuarius bennetti	Dwarf Cassowary	Frugivore (1,2)	Fr									
Centropus phasianinus	Pheasant Coucal	Large insects, frogs, lizards, eggs and young of birds (1).	In									
Ceyx azureus	Azure Kingfisher	Aquatic insec, other invertebrates and fish (1)	In	Yes		10			10	In	Yes
Ceyx lepidus	Moluccan Dwarf Kingfisher	Insectivore (1, 2)	In	Yes		8			8	In	Yes
Ceyx pusillus	Little Kingfisher	Mostly insects and their larvae, crustaceans, reptiles (1).	In									
Chaetorhynchus papuensis	Drongo Fantail	Insectivore (1, 2)	In									
Chalcophaps indica	Common Emerald Dove	Primarily fruit and seeds with some invertebrates (1).	Fr-In									
Chalcophaps stephani	Stephan's Emerald Dove	Frugivore (1,2)	Fr	Yes	5				5	Fr	Yes
Charmosyna papou	Papuan Lorikeet	Feeds on nectar, pollen, possibly flowers (1).	Ne									
Charmosyna placentis	Red-flanked Lorikeet	Apparently mainly or exclusively flowers and their nectar and pollen (1). Nectarivore (2).	Ne									
Charmosyna rubronotata	Red-fronted Lorikeet	Feeds on pollen, nectar, flowers and seeds (1).	Ne									
Charmosyna wilhelminae	Pygmy Lorikeet	Feeds on nectar and pollen (1).	Ne									
Chlamydera lauterbachi	Yellow-breasted Bowerbird	Mostly fruits, also insects (1).	Fr-In									
Chrysococcyx minutillus	Little Bronze Cuckoo	Insect, mainly caterpillars, also beetles and bugs (1).	In									
Chrysococcyx ruficollis	Rufous-throated Bronze Cuckoo	Insects, mainly caterpillars (1).	In									
Cicinnurus regius	King Bird-of-paradise	Fruits and arthropods; no information on relative proportions of each (1).	Fr-In									
Cinnyris jugularis	Olive-backed Sunbird	Insect, also nectar (1). Nectarivore (2).	In-Ne									
Clytoceyx rex	Shovel-billed Kookaburra	Earthworms, insect and their larvae, said also to take small vertebrates (1).	In									
Clytomyias insignis	Orange-crowned Fairywren	Insectivore (1).	In	Yes		3	1		4	In	Yes
Cnemophilus loriae	Loria's Satinbird	Mostly fruits; earthworms also eaten (1). Frugivore (2)	Fr-In									
Cnemophilus macgregorii	Crested Satinbird	Only fruits recorded (1).	Fr	Yes	3		1		4	Fr	Yes
Colluricincla megarhyncha	Little Shrikethrush	Insectivore (1, 2)	In	Yes	3	10	3	1	17	In	Yes
Columba vitiensis	Metallic Pigeon	Mainly various fruits, grains, seeds and berries (1).	Fr									
Coracina boyeri	Boyer's Cuckooshrike	Eats mainly fruit, especially figs, but takes also insect (1).	Fr-In									

Coracina caeruleogrisea	Stout-billed Cuckooshrike	Eats large insects, including weevils (Curculionidae) and other beetles (Coleoptera), also cockroaches (Blattodea) and lepidopteran larvae; fruit and small lizards also taken (1). Omnivore (2).	In								
Coracina incerta	Black-shouldered Cicadabird	Arthropods, including caterpillars; also fruit (1). Insectivore (2)	In								
Coracina longicauda	Hooded Cuckooshrike	Mainly insects; a little fruit also taken; small vertebrates recorded (1).	In								
Coracina melas	Black Cicadabird	Feeds mainly on insects; also takes fruit (1).	In	Yes		3	1		4	In	Yes
Coracina montana	Black-bellied Cuckooshrike	Eats fruit, including figs (Ficus); also insects, including caterpillars (1) Insectivore (2)	Fr-In	Yes		8	2		10	In	No
Coracina papuensis	White-bellied Cuckooshrike	Mainly larger insect (1).	In								
Coracina schisticeps	Grey-headed Cuckooshrike	Eats mainly fruit, also insect (1). Frugivore (2).	Fr-In								
Coracina tenuirostris	Common Cicadabird	Eats mainly insects; sometimes takes fruit and seeds (1). Omnivore (2).	In								
Corvus tristis	Grey Crow	Feeds mainly on fruits; also takes insects (1). Frugivore (2).	Fr-In								
Cracticus cassicus	Hooded Butcherbird	Feeds on large insects, and fruit; small vertebrates also taken (1).	Fr-In								
Cracticus quoyi	Black Butcherbird	Invertebrates, mainly insects; also small vertebrates, e.g. small lizards and snakes, frogs, small mammals and birds, small crabs and fish (1). Omnivore (2)	In								
Crateroscelis murina	Rusty Mouse-warbler	Insectivore (1, 2)	In	Yes		8	3		11	In	Yes
Crateroscelis nigrorufa	Bicolored Mouse-warbler	No details of diet (1). Congeners mostly insectivorous (3).	In								
Crateroscelis robusta	Mountain Mouse-warbler	Insectivore (1, 2)	In	Yes	3	9	9	1	22	Fr-In	No
Cyclopsitta diophthalma	Double-eyed Fig Parrot	Fig seeds principal food item (1).	Fr								
Cyclopsitta gulielmitertii	Orange-breasted Fig Parrot	Figs and other fruit, as well as whole small fruits, nectar from flowers and occasionally insects (1).	Fr								
Dacelo gaudichaud	Rufous-bellied Kookaburra	Arthropods, also small vertebrates (1).	In	Yes		4			4	In	Yes
Daphoenositta miranda	Black Sittella	Largely insectivorous (1).	In								
Dicaeum geelvinkianum	Red-capped Flowerpecker	Fruits, and possibly nectar and pollen; also spiders (1). Frugivore (2).	Fr								
Dicrurus bracteatus	Spangled Drongo	Insectivore (1, 2)	In								
Diphyllodes magnificus	Magnificent Bird-of-paradise	Omnivore (1, 2).	Fr-In								
Ducula chalconota	Rufescent Imperial Pigeon	Frugivore (1,2)	Fr								
Ducula pinon	Pinon's Imperial Pigeon	Frugivore (1,2)	Fr								
Ducula rufigaster	Purple-tailed Imperial Pigeon	Frugivore (1,2)	Fr								
Ducula zoeae	Zoe's Imperial Pigeon	Frugivore (1,2)	Fr								
Eclectus roratus	Eclectus Parrot	Fruits, berries, nuts; nectar, leaf buds and blossoms (1).	Fr								
Epimachus fastosus	Black Sicklebill	Fruits and animals in about equal proportion (1). Insectivore (2)	Fr-In								

Epimachus meyeri	Brown Sicklebill	Fruits, also arthropods and small vertebrates, in fairly equal proportions (1).	Fr-In						
Erythropitta erythrogaster	Red-bellied Pitta	Insectivore (1, 2)	In						
Eudynamys scolopaceus	Asian Koel	Omnivorous, consuming a variety of insects, caterpillars, eggs, small vertebrates, and fruits (1).	Fr-In						
Eugerygone rubra	Garnet Robin	Insectivore (1, 2)	In						
Eulacestoma nigropectus	Wattled Ploughbill	Insectivore (1, 2)	In						
Eurystomus orientalis	Oriental Dollarbird	Exclusively flying insects (1).	In						
Garritornis isidorei	Papuan Babbler	Range of arthropods; small reptiles also taken (1).	In	Yes	10		10	In	Yes
Geoffroyus geoffroyi	Red-cheeked Parrot	Frugivore (1,2)	Fr						
Geoffroyus simplex	Blue-collared Parrot	Seeds found in stomachs, but regularly visits flowering trees presumably for nectar and blossoms (1). Frugivore (2).	Fr						
Gerygone chloronota	Green-backed Gerygone	Insectivore (1).	In						
Gerygone chrysogaster	Yellow-bellied Gerygone	Insectivorous, but no details of prey (1).	In	Yes	3	1	4	In	Yes
Gerygone palpebrosa	Fairy Gerygone	Insectivore (1, 2)	In						
Gerygone ruficollis	Brown-breasted Gerygone	Insectivore (1, 2)	In						
Grallina bruijnii	Torrent-lark	Invertebrate; small lizards occasionally (1).	In						
Gymnophaps albertisii	Papuan Mountain Pigeon	Frugivore (1,2)	Fr						
Henicophaps albifrons	New Guinea Bronzewing	Takes fallen fruits, grubs and insect (1).	Fr						
Heteromyias albispecularis	Ashy Robin	Insectivore (1).	In	Yes	2	4	6	Fr-In	No
Ifrita kowaldi	Blue-capped Ifrit	Insects, including beetles; occasionally soft fruit (1). Insectivore (2).	In	Yes	4		4	In	Yes
Lalage atrovirens	Black-browed Triller	Fruits and arthropods; no information on relative proportions of each (1).	Fr-In						
Leptocoma sericea	Black Sunbird	Various arthropods, fruit and nectar (1). Nectarivore (2)	In-Ne						
Loboparadisea sericea	Yellow-breasted Satinbird	Frugivore (1,2)	Fr						
Lonchura tristissima	Streak-headed Mannikin	Seeds of grasses (1).	Gr	Yes	3 (seed)	1 (most. seeds)	4	Gr	Yes
Lophorina superba	Superb Bird-of-paradise	Fruits and variety of arthropods; proportions vary seasonally, from nearly all arthropods to almost entirely fruits (1). Omnivore (2).	Fr-In						
Loriculus aurantiifrons	Orange-fronted Hanging Parrot	Nectar, fruits, buds, flowers and seeds (1).	Ne						
Lorius lory	Black-capped Lory	Flowers for their pollen and nectar, also fruits and small insects (1). Nectarivore (2).	Ne						
Machaerirhynchus flaviventer	Yellow-breasted Boatbill	Insectivore (1, 2)	In						
Machaerirhynchus nigripectus	Black-breasted Boatbill	Insectivore (1, 2).	In						
Macropygia amboinensis	Slender-billed Cuckoo-Dove	Frugivore (1,2)	Fr						
Macropygia nigrirostris	Bar-tailed Cuckoo-Dove	Frugivore (1,2)	Fr						

Malurus alboscapulatus	White-shouldered Fairywren	Arthropods (1).	In								
Manucodia chalybatus	Crinkle-collared Manucode	Frugivore (1,2)	Fr								
Megapodius decollatus	New Guinea Scrubfowl	No information available, but presumably omnivorous (1).	Fr-In								
Melampitta lugubris	Lesser Melampitta	Insects, also vertebrates and fruits (1).	In								
Melanocharis longicauda	Mid-mountain Berrypecker	Small berries, likely to take also insect (1).	Fr-In								
Melanocharis nigra	Black Berrypecker	Small berries, also insect (1). Frugivore (2)	Fr-In	Yes	7	7	5	1	20	Fr-In	Yes
Melanocharis striativentris	Streaked Berrypecker	Known to take small berries (1). Frugivore (2).	Fr	Yes	6		3		9	Fr	Yes
Melanocharis versteri	Fan-tailed Berrypecker	Feeds on small berries and arthropods (1). Frugivore (2).	Fr-In	Yes	12	3	19	1	35	Fr-In	Yes
Melanorectes nigrescens	Black Pitohui	Insects and fruit, occasionally seeds (1).	Fr-In	Yes		10			10	In	No
Melidectes belfordi	Belford's Melidectes	Arthropods, mainly insect, including large ants and beetles, also nectar, fruit, also nectar and probably pollen (1).	In-Ne	Yes			4		4	Fr-In	No
Melidectes fuscus	Sooty Melidectes	Arthropods, nectar, pollen and fruit (1). Mostly insectivorous (4).	In-Ne	Yes		3	8	3	14	In-Ne	Yes
Melidectes princeps	Long-bearded Melidectes	No info (1). 60% food samples included insect only, 40% included insect and nectar (3).	In-Ne	Yes		8		5	13	In-Ne	Yes
Melidectes rufocrissalis	Yellow-browed Melidectes	Diet includes insects, nectar and fruit; commonly seen in flowering or fruiting trees (1). Omnivore (2)	Fr-In								
Melidectes torquatus	Ornate Melidectes	Diet invertebrates, mainly insect and their larvae, also nectar and small fruits (1). Omnivore (2)	Fr-In								
Melidora macrorrhina	Hook-billed Kingfisher	Large insects, also frogs (1). Insectivore (2).	In								
Melilestes megarhynchus	Long-billed Honeyeater	Small arthropods, small lizards, nectar, occasionally fruit (1). Insectivore (2).	In-Ne	Yes		3	1	1	5	In-Ne	Yes
Meliphaga analoga	Mimic Honeyeater	Omnivore (1, 2). Insect and fruits taken (3).	Fr-In	Yes	5	4	8	1	18	Fr-In	Yes
Meliphaga aruensis	Puff-backed Honeyeater	Diet includes fruit, seeds, and arthropods; probably also nectar (1). Omnivore (2).	Fr-In								
Meliphaga montana	Forest Honeyeater	Fruit, seed, and arthropods, probably also nectar, but said rarely, if ever (1).	Fr-In								
Meliphaga orientalis	Mountain Honeyeater	Diet nectar, arthropods (mainly insects), sometimes fruit (1). Omnivore (2).	In-Ne								
Melipotes fumigatus	Common Smoky Honeyeater	Predominantly frugivores, but insect and some floral parts also taken, not known to eat nectar (1). Frugivore (2).	Fr-In	Yes	2	2	4		8	Fr-In	Yes
Merops ornatus	Rainbow Bee-eater	Insectivore (1).	In								
Microdynamis parva	Dwarf Koel	Fruit (1).	Fr								
Microeca flavovirescens	Olive Flyrobin	Insectivore (1).	In								
Microeca griseoceps	Yellow-legged Flyrobin	Insects and other small arthropods (1).	In								
Microeca papuana	Canary Flyrobin	Insectivore (1, 2)	In	Yes		6	5		11	Fr-In	No
Micropsitta bruijnii	Red-breasted Pygmy Parrot	Lichen, fungi and moss with perhaps insects (Lexicon of Parrots).	In-Ne								
Micropsitta pusio	Buff-faced Pygmy Parrot	Lichen, bark fungus, and probably termites; insect remains, tiny black seeds, yellow fruit flesh and flowers in stomachs (1).	In-Ne	Yes				10	10	In-Ne	Yes

Mino anais	Golden Myna	Only fruit (1).	Fr	Yes		4	2		6	In	No
Mino dumontii	Yellow-faced Myna	Mainly fruit, also insects (1).	Fr-In	Yes		9	5		14	Fr-In	Yes
Monachella muelleriana	Torrent Flyrobin	Insectivore (1).	In	Yes		3	1		4	In	Yes
Monarcha frater	Black-winged Monarch	Food items not well known, mostly small to medium-size invertebrates (1), Insectivorous (2)	In								
Monarcha rubiensis	Rufous Monarch	Largely unknown, but mainly invertebrates (1).	In								
Myiagra alecto	Shining Flycatcher	Largely insectivorous; some fruit, may be taken (1). Insectivore (2).	In	Yes		9	1		10	In	Yes
Myzomela rosenbergii	Red-collared Myzomela	Primarily nectar, also small arthropods, ocassionaly smam fruits (1). Nectarivore (2).	In-Ne	Yes		1		3	4	In-Ne	Yes
Neopsittacus musschenbroekii	Yellow-billed Lorikeet	Pollen, nectar, flowers, small fruits and berries. Also may feed on insect (1).	Ne								
Neopsittacus pullicauda	Orange-billed Lorikeet	Eats pollen, nectar, flowers, fruits and less often, seeds (1).	Ne								
Oedistoma iliolophus	Dwarf Longbill	Feeds on insect, nectar and sometimes fruits (1). Insectivore (2).	In-Ne	Yes		2	1	1	4	In-Ne	Yes
Oreocharis arfaki	Tit Berrypecker	Stomach contents included entirely small fruits (1). Frugivore (2).	Fr								
Oreopsittacus arfaki	Plum-faced Lorikeet	Nectar and perhaps pollen (1). Nectarivore (2).	Ne								
Oriolus szalayi	Brown Oriole	Fruits, insect, also some grass seeds and probably nectars (1). Frugivore (2).	Fr-In								
Ornorectes cristatus	Crested Pitohui	Insectivore (1, 2)	In								
Otidiphaps nobilis	Pheasant Pigeon	Seeds and fallen fruit (1). Frugivore (2).	Fr								
Pachycare flavogriseum	Goldenface	Insectivore (1, 2)	In								
Pachycephala hyperythra	Rusty Whistler	Insectivore (1, 2)	In	Yes		3	5		8	Fr-In	No
Pachycephala modesta	Brown-backed Whistler	Insects, occasionally fruits (1).	In	Yes		3	1		4	In	Yes
Pachycephala schlegelii	Regent Whistler	Insectivore (1, 2)	In	Yes		6	10		16	Fr-In	No
Pachycephala simplex	Grey Whistler	Mainly insects, spiders; occasionally seeds (1). Insectivore (2).	In	Yes		3	1		4	In	Yes
Pachycephala soror	Sclater's Whistler	Insectivore (1, 2)	In	Yes		1	2		4	Fr-In	No
Pachycephalopsis poliosoma	White-eyed Robin	Insectivore (1, 2)	In	Yes		10			10	In	Yes
Paradisaea minor	Lesser Bird-of-paradise	Mostly fruits, also arthropods (1). Diet includes fruits and animal prey (5).	Fr-In								
Paramythia montium	Crested Berrypecker	Almost entirely small fruits, insects recorded rarely in stomach (1).	Fr	Yes	9		4		13	Fr	Yes
Peltops blainvillii	Lowland Peltops	Mainly flying insects (1).	In								
Peltops montanus	Mountain Peltops	Insectivore (1, 2)	In								
Peneothello bimaculata	White-rumped Robin	Insectivore (1, 2)	In	Yes		8	2		10	In	Yes
Peneothello cyanus	Slaty Robin	Insectivore (1, 2)	In	Yes		8	14		22	Fr-In	No
Peneothello sigillata	White-winged Robin	Insectivore (1, 2)	In	Yes		7	13		20	Fr-In	No
Philemon buceroides	Helmeted Friarbird	Eats nectar, fruit, insects and other invertebrates (1).	In-Ne								

Philemon meyeri	Meyer's Friarbird	Fruit, nectar and insect (1).	In-Ne								
Phylloscopus maforensis	Island Leaf Warbler	Insectivore (1, 2)	In								
Pitohui dichrous	Hooded Pitohui	Mainly fruit, including small figs (Ficus); some insects and grass seeds (1). Insectivore (2)	Fr-In	Yes			4		4	Fr-In	Yes
Pitohui kirhocephalus	Northern Variable Pitohui	Insects and fruit (1). Insectivore (2).	In	Yes		10			10	In	Yes
Pitta sordida	Hooded Pitta	Insects of many kinds (1). Insectivore (2).	In								
Podargus ocellatus	Marbled Frogmouth	Insectivore (1, 2)	In								
Poecilodryas albonotata	Black-throated Robin	Insect (1), Insectivorous (2)	In								
Poecilodryas hypoleuca	Black-sided Robin	Insectivore (1).	In	Yes		3	1		4	In	Yes
Probosciger aterrimus	Palm Cockatoo	Feed on seeds, nuts, berries (1).	Fr								
Pseudeos fuscata	Dusky Lory	Feeds on flowers, fruits and insects (1).	Fr-In								
Pseudorectes ferrugineus	Rusty Pitohui	Insects and fruit (1). Omnivore (2).	Fr-In								
Psittacella brehmii	Brehm's Tiger Parrot	Frugivore (1,2)	Fr								
Psittacella picta	Painted Tiger Parrot	Eats seeds, berries, and fruits (1).	Fr								
Psittaculirostris edwardsii	Edwards's Fig Parrot	Feeds on fruits, nectar and possibly insects (1).	Fr								
Psitteuteles goldiei	Goldie's Lorikeet	Pollen, nectar, flowers and berries (1).	Ne								
Psittrichas fulgidus	Pesquet's Parrot	Highly specialised frugivore (Ficus; 1).	Fr								
Pteridophora alberti	King of Saxony Bird-of-paradise	Mostly fruits, also insects and spiders (1).	Fr-In								
Ptilinopus coronulatus	Coroneted Fruit Dove	Frugivorous, feed on variety of fruits, particularly figs (1).	Fr								
Ptilinopus iozonus	Orange-bellied Fruit Dove	Feed on fruits, mainly on figs (84%; 1).	Fr								
Ptilinopus magnificus	Wompoo Fruit Dove	Frugivore (1,2)	Fr								
Ptilinopus ornatus	Ornate Fruit Dove	Frugivore (1,2)	Fr								
Ptilinopus perlatus	Pink-spotted Fruit Dove	Feed on fruit, especially figs (1).	Fr								
Ptilinopus pulchellus	Beautiful Fruit Dove	Frugivore (1,2)	Fr								
Ptilinopus rivoli	White-bibbed Fruit Dove	Frugivore (1,2)	Fr								
Ptilinopus superbus	Superb Fruit Dove	Frugivore (1,2)	Fr								
Ptiloprora guisei	Rufous-backed Honeyeater	Predomiantly arthropods, also fruit, seeds and nectar (1). Omnivore (2).	Fr-In	Yes		2	2		4	Fr-In	Yes
Ptiloprora perstriata	Grey-streaked Honeyeater	Diet includes arthorpods, fruit and nectar (1).	Fr-In	Yes	4	9	10	2	25	Fr-In	Yes
Ptiloris magnificus	Magnificent Riflebird	Fruits and arthropods; overall a greater proportion of arthropods eaten, but relative proportions vary seasonally (1). Insectivore (2)	Fr-In	Yes		2	4		6	Fr-In	Yes
Ptilorrhoa caerulescens	Blue Jewel-babbler	Insectivore (1).	In	Yes		10			10	In	Yes
Ptilorrhoa castanonota	Chestnut-backed Jewel-babbler	Insects; possibly also small frogs (1). Insectivore (2).	In								
Ptilorrhoa leucosticta	Spotted Jewel-babbler	Insectivore (1, 2).	In								
Pycnopygius ixoides	Plain Honeyeater	Diet small fruits and nectar (1).	Fr								

Rallicula forbesi	Forbes's Forest Rail	Invertebrates, small vertebrates, and seeds (1).	In								
Reinwardtoena reinwardti	Great Cuckoo-Dove	Feeds on a variety of small seeds and fruit (1). Frugivore (2).	Fr								
Rhagologus leucostigma	Mottled Berryhunter	Insect, berries (1). Omnivore (2). 80% included insect and fruits in equal proportion (3).	Fr-In	Yes	1	1	7		9	Fr-In	Yes
Rhipidura albolimbata	Friendly Fantail	Insectivore (1, 2).	In	Yes		14	2		16	In	Yes
Rhipidura atra	Black Fantail	Insectivore (1,2)	In	Yes		30	4		34	In	Yes
Rhipidura brachyrhyncha	Dimorphic Fantail	Insectivore (1, 2).	In	Yes		3	3		6	Fr-In	No
Rhipidura leucothorax	White-bellied Thicket Fantail	Insectivore (1).	In								
Rhipidura rufidorsa	Rufous-backed Fantail	Insectivore (1).	In	Yes		10			10	In	Yes
Rhipidura rufiventris	Northern Fantail	Insectivore (1, 2).	In	Yes		4			4	In	Yes
Rhipidura threnothorax	Sooty Thicket Fantail	Small insects (1). Insectivore (2).	In	Yes		3	1		4	In	Yes
Rhyticeros plicatus	Blyth's Hornbill	Mainly of fruits – especially figs (Ficus) - occasionally supplemented with insect (1).	Fr								
Saxicola caprata	Pied Bush Chat	Insectivore (3).	In								
Scolopax rosenbergii	New Guinea Woodcock	Two stomach analysed yielded caterpillars and moth pupae (1).	In								
Scythrops novaehollandiae	Channel-billed Cuckoo	Fruit, especially figs; also insects (1).	Fr-In								
Sericornis arfakianus	Grey-green Scrubwren	Poorly known, likely to be insectivorous (1). Insectivorous (1).	In	Yes		4			4	In	Yes
Sericornis nouhuysi	Large Scrubwren	Insectivore (1, 2).	In	Yes		6	12		18	Fr-In	No
Sericornis papuensis	Papuan Scrubwren	Insectivore (1, 2).	In	Yes		2	6		8	Fr-In	No
Sericornis perspicillatus	Buff-faced Scrubwren	Insectivore (1, 2).	In	Yes		9	13		22	Fr-In	No
Sericornis spilodera	Pale-billed Scrubwren	Insectivore (1, 2).	In	Yes		1	3		4	In	Yes
Syma megarhyncha	Mountain Kingfisher	Insects, larvae, and small lizards (1). Insectivore (2).	In	Yes		3	1		4	In	Yes
Syma torotoro	Yellow-billed Kingfisher	Insect and small lizzards (when in forest), small fish (in mangrove habitats (1).	In	Yes		10			10	In	Yes
Symposiachrus axillaris	Black Monarch	Insectivore (1,2)	In								
Symposiachrus guttula	Spot-winged Monarch	Insectivore (1, 2)	In								
Symposiachrus manadensis	Hooded Monarch	Insectivore (1).	In								
Talegalla jobiensis	Collared Brushturkey	No information available, though considered to be probably omnivorous (1).	Fr-In								
Tanysiptera galatea	Common Paradise Kingfisher	Insectivore (1, 2).	In	Yes		8			8	In	Yes
Timeliopsis fulvigula	Olive Straightbill	Insectivore (1, 2).	In								
Todiramphus macleayii	Forest Kingfisher	Invertebrates (1).	In								
Toxorhamphus novaeguineae	Yellow-bellied Longbill	Insectivorous, taking also nectar but details not known (1).	In-Ne	Yes	2	7	1	1	11	In-Ne	Yes
Toxorhamphus poliopterus	Slaty-headed Longbill	Diet poorly known; feeds on arthropods and nectar (1). Nectarivore (2). Insect in 100% of samples, nectar in 40% of samples (3).	In-Ne	Yes		2	2	1	5	In-Ne	Yes

Tregellasia leucops	White-faced Robin	Insectivore (1, 2).	In	Yes		2	2		4	Fr-In	No
Trichoglossus haematodus	Coconut Lorikeet	Nectar and pollen (1). Nectarivore (2).	Ne								
Turdus poliocephalus	Island Thrush	Insectivore (1, 2).	In	Yes	2	1	2		5	Fr-In	No
Xanthotis flaviventer	Tawny-breasted Honeyeater	Mainly insects, also nectar and fruit, e.g. of figs (1). Insectivore (2).	In-Ne	Yes	2		1	2	5	In-Ne	Yes
Zoothera heinei	Russet-tailed Thrush	Worms and molluscs; also some fruit (1). Invertebrates and fruits (2).	Fr-In	Yes		2	2		4	Fr-In	Yes
Zosterops minor	Black-fronted White-eye	Insects and fruits; latter include various figs (1). Congeners mostly insectivorous (3).	In	Yes		3	1		4	In	Yes
Zosterops novaeguineae	Papuan White-eye	Stomach contents included spiders, insects, fruits and seeds (1).	In								

Figure S2. Relative importance of food types (plant material or arthropod's body parts) in food samples of birds partitioned into feeding guild according to literature and mist-netted in understory of Mt. Wilhelm elevational gradient. Note that we did not mist-net any representatives of some guilds at some elevational study sites (i.e. elevation is missing at axis X).

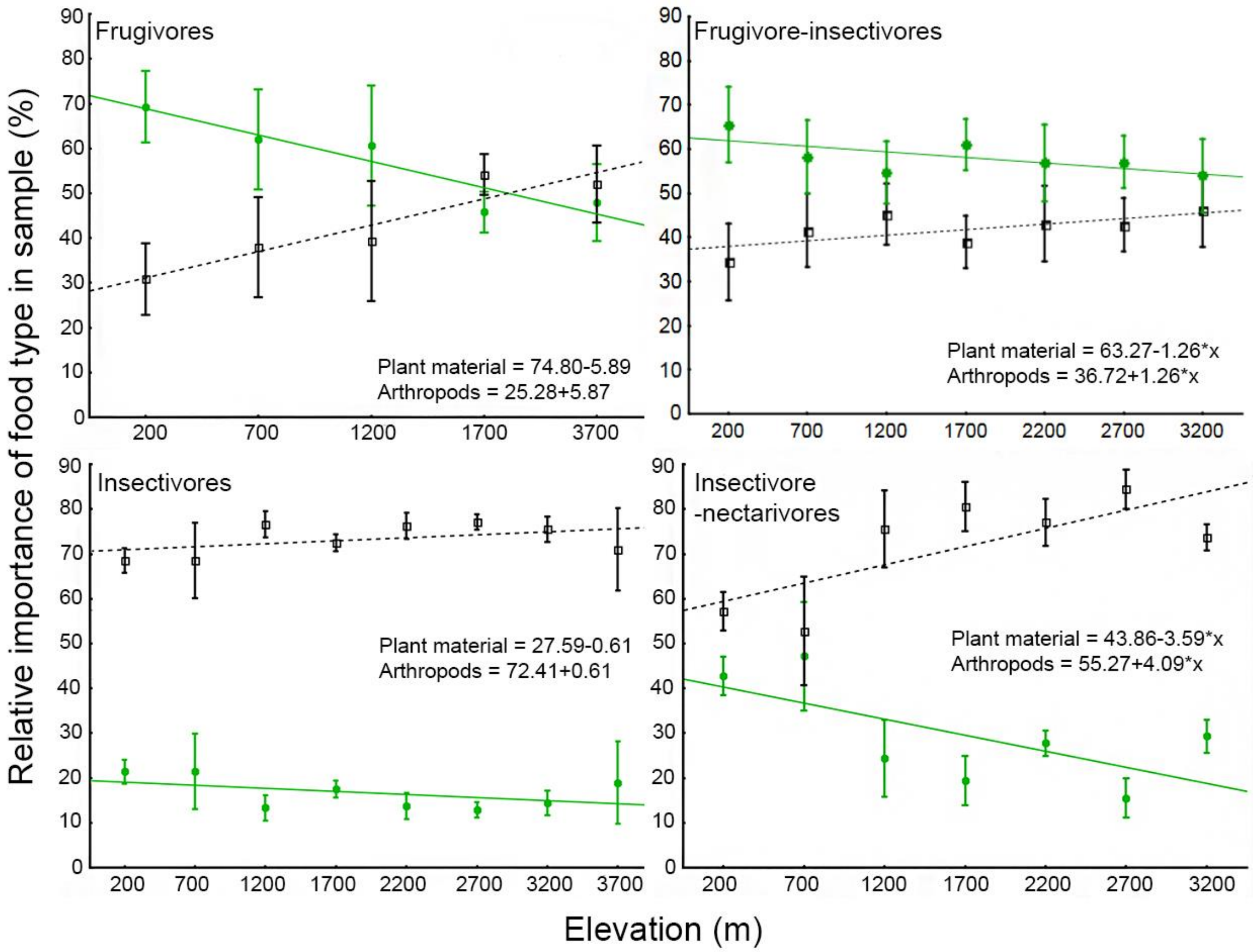

Figure S3. Randomised accumulation curves of food morphotypes taken by six bird species (with N ≥ 9 from the same elevation). Sample = regurgitated food from an individual bird. CollMega = *Colluricincla megarhyncha*, 200m; MelaNigr = *Melanocharis nigra*, 700m; CratRobu = *Crateroscelis robusta*, 2700m; MelaVers = *Melanocharis versteri*, 1700m; SeriPers = *Sericornis perspicillatus*, 2700m; RhipAtra = *Rhipidura atra*.

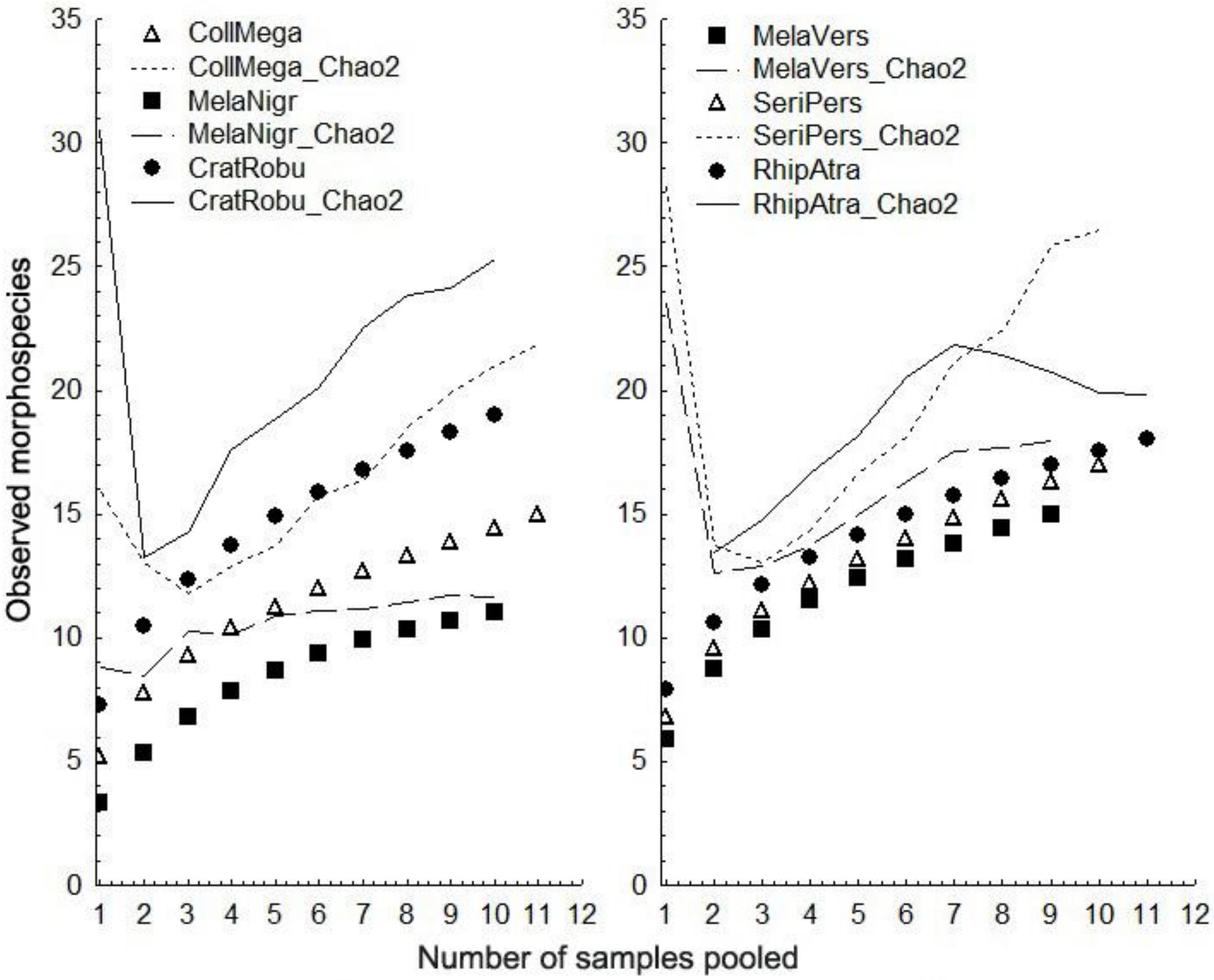

Figure S4. Relative species richness of birds along Andean (a) and Costa Rica (b) elevational gradients. Data extracted from Terborgh 1977 and Blake et al. 2000. Elevational study sites are shown approximately. (Terborgh, J. 1977. Bird species diversity on an Andean elevational gradient. Ecology **58**:1007-1019.; Blake, G. John, Loiselle and A. Bette 2000. Diversity of birds along an elevational gradient in the Cordillera Central, Costa Rica. The Auk **117**: 663-686.)

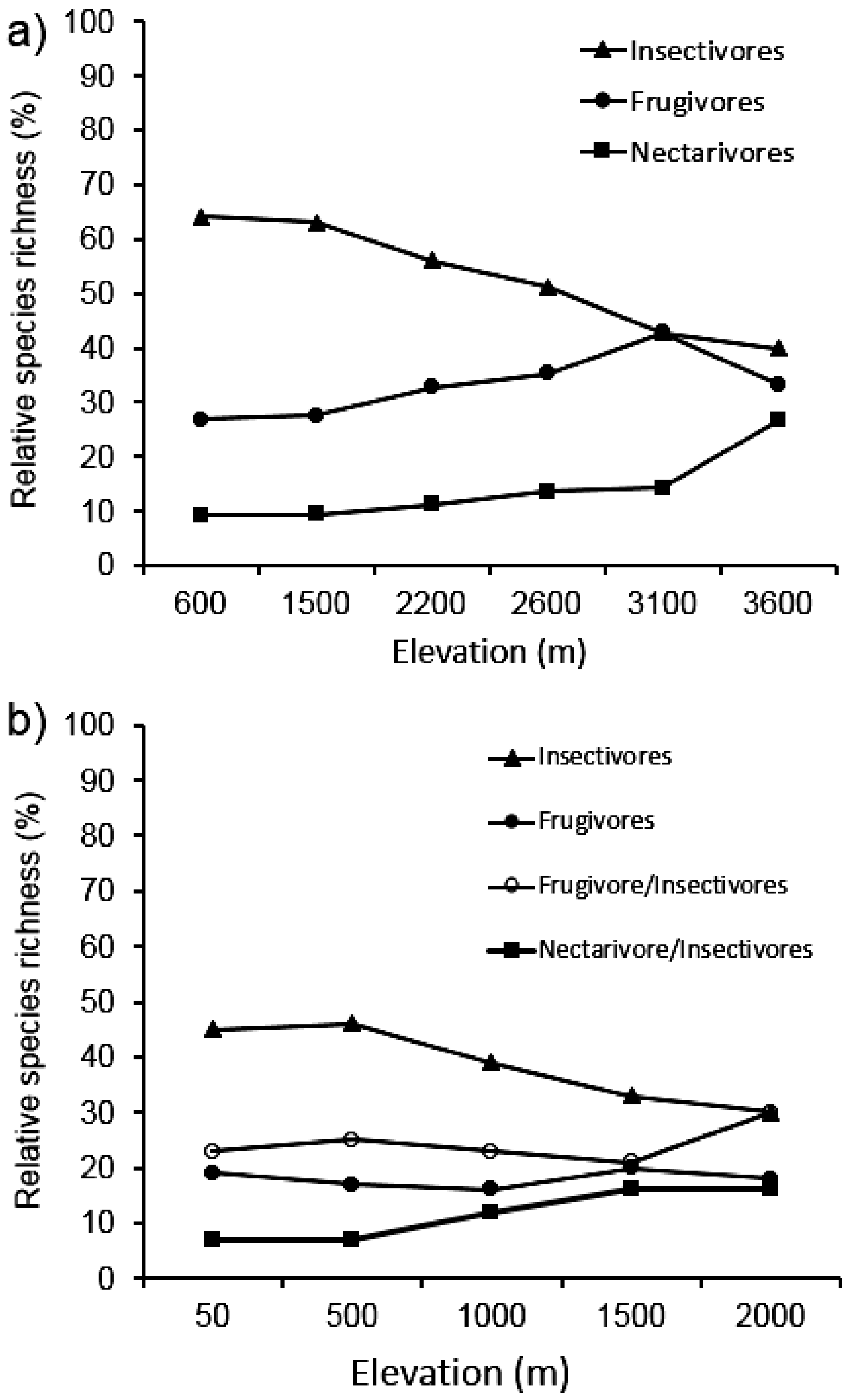

Supplement: Supplementary Materials [file srep44018-s1.pdf]
